# Endocannabinoids as Guardians of Metastasis

**DOI:** 10.3390/ijms17020230

**Published:** 2016-02-10

**Authors:** Irmgard Tegeder

**Affiliations:** Institute of Clinical Pharmacology, University Hospital Frankfurt, 60590 Frankfurt am Main, Germany

**Keywords:** endocannabinoids, anandamide, 2-arachidonoylglycerol, orphan G-protein coupled receptor, immune cells, angiogenesis

## Abstract

Endocannabinoids including anandamide and 2-arachidonoylglycerol are involved in cancer pathophysiology in several ways, including tumor growth and progression, peritumoral inflammation, nausea and cancer pain. Recently we showed that the endocannabinoid profiles are deranged during cancer to an extent that this manifests in alterations of plasma endocannabinoids in cancer patients, which was mimicked by similar changes in rodent models of local and metastatic cancer. The present topical review summarizes the complexity of endocannabinoid signaling in the context of tumor growth and metastasis.

## 1. Introduction

**Figure 1:**
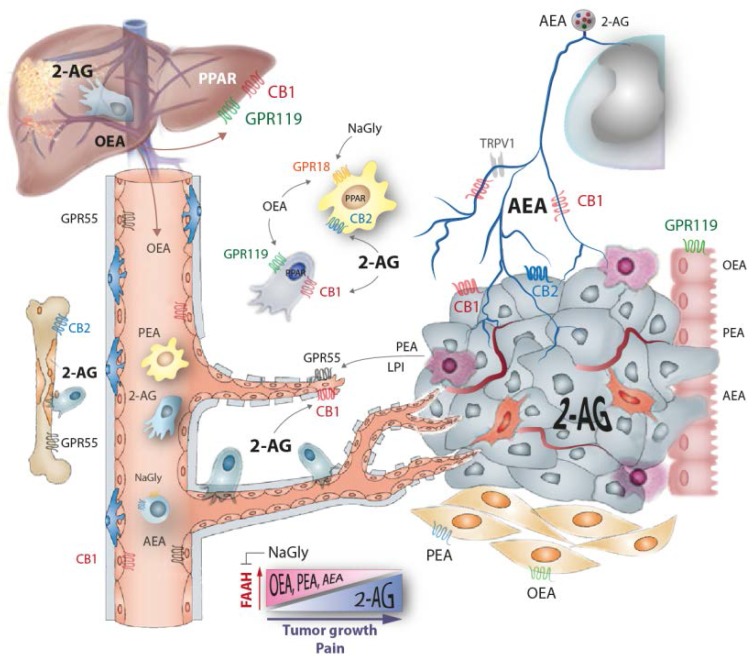
Tumor growth causes an increase of 2-arachidonoylethanolamide (2-AG) in tumor tissue and plasma and a decrease of ethanolamide endocannabinoids anandamide (AEA), oleoylethanolamide (OEA), palmitoylethanolamide (PEA) because the tumor displaces normal tissue and increases fatty acid amide hydrolase (FAAH) expression. It is likely that the tumor itself mainly secretes 2-AG. The endocannabinoids have diverse effects on cannabinoid receptors including the typical cannabinoid receptor 1 and 2 (CB1, CB2), the orphan G-protein coupled receptors (GPRs) 18, 55, 92 and 119 and peroxisome proliferator activated receptor (PPAR) gamma and alpha, and AEA also acts on transient receptor potential family V type 1 (TRPV1), resulting in complex regulation of tumor growth, metastasis, angiogenesis, polarization of tumor-associated macrophages and dendritic cells, T-cell activation and cancer pain. Treatment with exo- and endogenous cannabinoids reduced cancer growth in several rodent models and cell culture experiments [25,26,27,28,29,30,31], but CB2 expression of the tumor itself has been recently associated with poor prognosis in breast cancer [32].

Endocannabinoids (eCBs) constitute a growing number of lipid signaling molecules, the most popular being anandamide (AEA) and 2-arachidonoylglycerol (2-AG). They are involved in cancer pathophysiology in several ways (Figure 1), including tumor growth and progression [1], immune (in)tolerance, inflammation [2], nausea [3] and cancer pain [4,5]. Our recent work [6] revealed that the endocannabinoid profiles are deranged during cancer, particularly in metastatic cancer, to an extent that this manifests in alterations of plasma endocannabinoids in cancer patients, which was mimicked by similar changes in rodent models of local and metastatic cancer, suggesting that the monitoring of endocannabinoid profiles might be useful for assessing the individual course of the disease and, possibly, that the derangement of the profiles plays a functional role for cancer progression, potentially giving rise to supportive therapeutic interventions.

## 2. Rise of 2-AG in the Tumor Environment and in Plasma

Endocannabinoids are produced in several peripheral tissues resulting in cell-type and location-specific profiles so that the eCB pattern in the tumor microenvironment depends on the tumor’s origin and site of primary growth and metastasis. We showed that tumor growth is associated with an increase of 2-arachidonoylglycerol (2-AG) both at the site of the primary tumor and in plasma [6]. It steadily increased over the course of cancer development and metastasis, suggesting that the growing tumor and circulating metastatic tumor cells secrete large amounts of 2-AG, sufficient enough to manifest in high plasma concentrations. The 2-AG increase is likely contributed by activated immune cells, which are a major source of 2-AG in the periphery at sites of inflammation [7,8]. In the tumor microenvironment 2-AG elicits CB2 receptor signaling of invading immune cells, which may trigger a phenotypic switch from aggressive to tumor-tolerant cells [9] and polarization towards the tumor-helping M2-like macrophages, such as ”tumor associated macrophages” (TAMs) [10]. These TAMs promote tumor invasiveness and metastasis by releasing metalloproteinases and angiogenic factors [11].

One of the M2-derived pro-angiogenic factors is palmitoylethanolamide (PEA) which acts as an agonist of endothelial GPR55 receptors [12]. GPR55 is an untypical cannabinoid receptor, which elicits Rho, Rac and CD42 signaling [13], converging on the regulation of cancer and endothelial cell migration [14] and tube formation [15]. GPR55 is also activated by 2-AG-ether, a precursor of 2-AG, which is also known as noladin ether and is likely directly produced and released by the tumor. Further agonists of GPR55 include lysophospholipids, e.g., lysophosphatidylinositol (LPI) [15,16]. These lipids are released by aggregating platelets [17] or produced extracellularly by secretory phospholipases A [18] or D [19]. The latter, also known as autotaxin, attaches to the cell surface of circulating immune or metastatic tumor cells and uses their membrane lipids as precursors for the production of lysophosphatidic acids (LPAs) [20,21], which then stimulate tumor cell migration [22] and angiogenesis via LPA receptors [23]. Vascular cells also express CB1 and GPR18, which mediate vasodilation on agonist binding [24] and thereby increase the tumor’s blood supply.

## 3. Loss of Ethanolamide Endocannabinoids in Tumor Environment and Plasma

Contrary to 2-AG, we have shown that ethanolamide endocannabinoids (eCBs) anandamide (AEA), oleoylethanolamide (OEA) and palmitoylethanolamide (PEA) decrease in the tumor microenvironment and in plasma [6], likely because the growing tumor displaces normal cells that produce these eCBs and destroys sensory neuronal fibers that are innervating the tumor [33]. These terminals are a major source of AEA in the tumor microenvironment, and as long as these terminals secrete AEA the tumor itself may suppress local cancer pain. The peripheral AEA pool is further contributed by epithelial cells, keratinocytes and muscle cells that all release AEA on demand [34]. AEA is a full agonist of all kinds of cannabinoid receptors that have been identified including CB1, CB2, the orphan GPRs 18, 55, 92 (= LPAR5) and 119, and the nuclear receptors PPAR gamma and alpha [35,36], and transient receptor potential (TRPV1) calcium channels [37]. Most importantly, it acts as an autocrine agonist of CB1 receptors of the peripheral nerves to control nociception [7] and as a CB2 agonist to resolve inflammation. Its decline in the tumor environment is associated with an increase of cancer pain and a deregulation of immune cells.

The 2-AG, OEA and PEA have a narrower spectrum of receptors than AEA [38], which also holds true for the exogenous cannabis constituents, tetrahydrocannabinol (THC) and cannabidiol (CBD). Like AEA, both OEA and PEA reciprocally decrease while the tumor mass and metastases increase [6]. All of these eCBs are primarily metabolized by fatty acid amide hydrolase (FAAH) which is upregulated in various types of cancer [25,39], suggesting that enhanced degradation contributes to the loss of production by normal cells. So far, FAAH antagonists have been considered as potential treatments for cancer pain in rodent models [4], but their potential effects on tumor growth and the anti-tumoral immune response have not been assessed. However, interestingly the weak FAAH inhibitor R-flurbiprofen, which leads to a resetting of normal eCB profiles in models of neuropathic pain and autoimmune disease [40,41], was previously shown to reduce tumor growth in transplant models in nude mice [42] and in colon or prostate cancer development in APC^min^ (Adenomatous Polyposis Coli, multiple intestinal neoplasia) and TRAMP (Transgenic Adenocarcinoma of the Mouse Prostate) mice [43,44]. Although R-flurbiprofen failed in phase II clinical studies of prostate cancer, its positive results may encourage testing of FAAH-based combi-treatments for pain and cancer [45].

The primary source of peripheral OEA is the (gut) epithelium, fat and liver [46,47]. It has been mainly considered as a satiety signal, and consequently as a protector against obesity and metabolic syndrome [47,48,49]. Peripheral PEA is likely mainly produced by stromal and immune cells [50]. Both OEA and PEA do not activate the typical CB1 and CB2 cannabinoid receptors, but act through the “orphan” cannabinoid GPRs and PPARs: OEA mainly through GPR119, PPAR gamma and alpha, and PEA mainly through GPR55, GPR18 and PPAR alpha [35,36]. GPR18 and 92 are highly expressed by immune cells including macrophages, T- and B-cells, and the primary full agonist N-arachidonoylglycine, NaGly, a metabolite of AEA [51,52], regulates immune functions and cell migration through these receptors [53,54], suggesting that both may contribute to the fine-tuning of the tumor-evoked immune response. NaGly is produced by many cells and acts as an endogenous inhibitor of FAAH and thereby increases AEA, OEA and PEA levels [55] and counteracts the tumor-mediated loss of these eCBs.

## 4. Cannabinoid Receptors of Tumor Cells

Depending on the tumor’s origin, the tumor cells themselves express CB1 and CB2, which has been reviewed elsewhere [56,57,58], and possibly GPR119, the latter particularly in tumors of epithelial origin. Cannabinoid-mediated tumor killing was shown to involve mostly CB1 signaling: one path converging on an increase of ceramides that leads to the endoplasmic reticulum and oxidative stress [1], other pathways converging on Akt, Erk or MAP kinase inhibition [59,60], AMPK-mediated autophagy [61], cell cycle inhibition [62], or still unknown receptors and signaling pathways [63]. The expression of CB1 was identified as a positive prognostic factor for disease-free survival in patients with tongue cancer [64] but not prostate cancer [65], although prostate cancer cells, like other cancer cells, are killed by CB1 or CB2 agonists *in vitro* [25,66,67,68]. CB2 expression has been recently associated with a poor prognosis in Her2/Neu-positive breast cancer, where its presence promoted pro-oncogenic signaling of Her2 at the level of the tyrosine kinase c-Src [32]. In contract, triple-negative breast cancer cells (estrogen receptor, progesterone receptor and Her2-negative) were killed by a CB2 agonist [69]. It is obvious from multiple studies that the co-expression of the cannabinoid receptor with receptors of the epidermal growth factor receptor (EGFR) or other growth factor families is crucial for the outcome because the formation of heteromers [32] or signaling crosstalk may reverse normal functions. While CB1 and CB2 are well studied in the context of cancer, GPR119 is still an “orphan”, although it is highly expressed in glandular tissue including intestine, pancreas and liver, and its activation leads to lipolysis, insulin secretion and reduction of food intake [70,71]. It is mainly activated by OEA and triggers the OEA-evoked satiety signals [70,71]. 

## 5. Oleoylethanolamide

OEA concentrations in the plasma of cancer patients were reduced initially but re-raised once local tumor growth turned into metastatic disease [6]. OEA levels were positively associated with the number of metastases in cancer patients, and particularly liver metastases caused an increase of OEA plasma levels, suggesting enhanced secretion from the metastatic liver [6]. To assess the physiologic relevance of OEA in the context of metastasis, we tested its effects in a migration assay *in vitro* [6]. High OEA concentrations that are not reached in plasma, but in normal tissue surrounding locally restricted tumors, inhibited tumor cell migration. Oppositely, low concentrations enhanced tumor cell proliferation and migration [6], suggesting that the local loss of OEA in the tumor microenvironment facilitates growth and metastasis and the increase of liver OEA secretion may be interpreted as an attempt to stop metastasis. However, despite this metastasis-driven increase, plasma levels remained low compared to those required to stop migration. OEA *per se* did not qualify as an independent marker of metastasis but might be indicative of individual progression.

The concentration-dependent opposing effects of low and high OEA may involve actions through GPR119 and PPARs resulting in opposite pro- or anti-migratory signaling pathways. Besides OEA, anandamide is a strong agonist of both PPAR alpha and gamma whereas 2-AG and PEA mainly act as agonists of PPAR alpha [36]. Tumor cells, immune cells and endothelial cells all express PPARs and both activators and inhibitors were shown to reduce cancer growth or migration [72,73,74]. Hence, by acting through PPARs, all endocannabinoids and exogenous cannabinoids may facilitate or inhibit growth with an unforeseeable outcome. Overall, the net effect of cannabinoid treatment in various models of cancer was a reduction of cancer development and growth [25,26,27,28,29,30,31,59]. However, the opposite was also observed [75,76,77]. The complexity of the endocannabinoid system in the tumor microenvironment of local and metastatic cancer complicates the development of anti-cancer drugs targeting the endogenous cannabinoid system. Nevertheless, FAAH inhibition may be a logical approach to restore normal eCB balances [78,79], whereas inhibition of monoacylglycerol lipase (MAGL) and abhydrolase domain containing 6 (ABHD6) which metabolize 2-AG would instead further shift the balance towards 2-AG. Consequently, both MAGL and ABDH6 inhibition produced a variable outcome [75,80,81], which, however, may depend on the tumor’s origin.

## 6. Exogenous Cannabinoids and Therapeutic Implications

The exogenous cannabinoids THC and cannabidiol (CBD) reduced tumor growth in animal models [27,31,82,83,84]. THC acts as an agonist of GPR18, CB1 and CB2 whereas cannabidiol is an antagonist of GPR55, and an agonist of GPR18 and GPR119. Cannabidol does not act through the typical CB1 and CB2 receptors. Hence, the combination of THC with CBD, currently available as oromucosal spray, may favorably combine anti-proliferative CB1-mediated effects and suppression of GPR55-mediated angiogenesis and reduction of cancer pain [85,86], and by acting through GPR18, immune cells may be stimulated to migrate towards and kill tumor cells.

THC-mediated activation of CB2-mediated silencing of macrophages may be a disadvantage in terms of the tumor growth, and in certain types of cancer, expression of CB2 was associated with a poor prognosis [32]. On the other hand, CB2 signaling would reduce the surrounding inflammation and, likely, the reduction of cancer pain contributes to a fortification of the immune system, raising the idea of a broader use of cannabinoids in cancer (pain) treatment.

However, exogenous cannabinoids cannot replace or restore endogenous cannabinoid profiles, suggesting that drugs targeting the degradation of ethanolamide endocannabinoids may have additional therapeutic value. In addition, the monitoring of individual endocannabinoid profiles over time may be useful for an assessment of disease progression and identification of patients who would likely profit from an eCB-directed therapy.

## Abbreviations

eCB, endocannabinoid; AEA, anandamide; OEA, oleoylethanolamide; PEA, palmitoylethanolamide; 2-AG, arachidonoylethanolamide; FAAH, fatty acid amide hydrolase; LPI, phosphatidylinositol; NaGly, N-arachidonoylglycine; GPR, G-protein coupled receptor; CB1, CB2, cannabinoid receptor 1 and 2; PPAR, peroxisome proliferator activated receptor; TRPV1, transient receptor potential family V type 1. THC, tetrahydrocannabinol; AMPK, adenosine monophosphate-activated kinase; MAGL, monoacylglycerol lipase; ABHD6, abhydrolase domain containing 6; EGFR epidermal growth factor receptor; Her2/Neu, human epidermal growth factor receptor 2, erb-B2.
